# Detecting nematodes in potato plants an explainable machine learning approach for detection of potato cyst nematode infections using hyperspectral imaging

**DOI:** 10.1016/j.plaphe.2025.100127

**Published:** 2025-10-14

**Authors:** Janez Lapajne, Nik Susič, Andrej Vončina, Barbara Gerič Stare, Nicole Viaene, Jonathan Van Beek, David Nuyttens, Saša Širca, Uroš Žibrat

**Affiliations:** a Plant Protection Department, Agricultural Institute of Slovenia, Hacquetova ulica 17, Ljubljana, 1000, Ljubljana, Slovenia; b Plant Sciences Unit, Flanders Research Institute for Agriculture, Fisheries, and Food, Burgemeester Van Gansberghelaan 96, Merelbeke, 9820, Belgium; c Department of Biology, Ghent University, Ledeganckstraat 35, 9000, Ghent, Belgium; d Technology and Food Science Unit, Flanders Research Institute for Agriculture, Fisheries, and Food, Burgemeester Van Gansberghelaan 115, Bus 1, Merelbeke, 9820, Belgium

**Keywords:** Hyperspectral imaging, Machine learning, Potato cyst nematodes, Potato, *Globodera*, *Solanum tuberosum*, Detection

## Abstract

Potato cyst nematodes pose a major threat to potato cultivation, with infestations often going undetected for years. Early and accurate detection is crucial for effective management, necessitating reliable, large-scale monitoring methods. Hyperspectral imaging shows great promise for non-invasive nematode detection, yet distinguishing between biotic (e.g., nematodes) and abiotic (e.g., drought) stressors remains a challenge. This study investigated the stress responses of potato plants to potato cyst nematodes *Globodera rostochiensis* and *G. pallida*, and water deficiency. We generated datasets to isolate and evaluate single and combined stressor effects on plant physiology and morphology. Various machine learning models and spectral processing techniques were applied to assess classification performance. Exploratory methods identified key spectral wavelengths, while statistical analyses evaluated the significance of physiological and morphological traits. Results showed that water deficiency was the dominant classification factor (F1 ​= ​0.95). The distinction between infected and non-infected plants reached F1 ​= ​0.70 in well-watered conditions and 0.80 in water-deficient plants. Distinguishing nematode species and inoculation levels yielded moderate accuracy (F1 ​= ​0.65–0.80), improving to 0.80 when combining biotic and abiotic stress. However, classifying multiple stress categories simultaneously reduced performance (F1 ​= ​0.58). These findings highlight the challenges of stressor separation and the potential of hyperspectral imaging for nematode detection. Further research is needed to refine classification models and validate findings under field conditions, facilitating the integration of hyperspectral imaging into precision agriculture.

## 1 Introduction

Plant-parasitic nematodes, particularly potato cyst nematodes (PCN; *Globodera* spp.), are important pests of potato, with estimated losses of 9 ​% of total potato production. Failure to implement control strategies for PCN can result in an almost complete loss of the crop [1]. Two *Globodera* species, *Globodera pallida* (Stone) Behrens and *G. rostochiensis* (Wollenweber) Behrens, are listed as quarantine pests in many countries of the world [2,3], and are recognized as A2 quarantine pests in the European Union (Annex II B) [4]. Quarantine pests are of great importance in agriculture and are officially monitored due to their rapid spread and damage potential (Commission Implementing Regulation (EU) 2022/1192).

Potato plants infected with *G. pallida* and/or *G. rostochiensis* show reduced plant growth and chlorosis, initially appearing as localized patches or hotspots in the field. If no control measures are taken, the infestation can spread over time and become a full-field problem emphasizing the need for early detection and spatially accurate monitoring for effective pest control [5].

Laboratory diagnostics are needed for accurate identification of PCN, usually by morphological and morphometric analysis of the cysts and juveniles [6] as well as PCR [7] or qPCR analyses [8]. Visual examination of plant roots for developing cysts is one of the simplest detection methods, but it is invasive and extremely impractical for large-scale use, as the cysts are only visible on the roots for a relatively short time before they fall off as they mature. Soil sampling and subsequent extraction of cysts is the conventional method for detecting PCN and estimating their density in potato fields, but this can miss early centers of infestation [6].

Remote sensing techniques offer a solution by enabling large-scale, early detection of nematodes. This approach has the potential to significantly reduce human intervention, enable objective and non-invasive monitoring and provide a cost-effective solution [9]. Hyperspectral imaging (HSI) is widely used [10] to detect biotic and abiotic stress in crops [11,12], including nematode-infected potato plants [13], due to its proven efficacy. The visible and near-infrared (VNIR) range is ideal for analyzing plant health, assessing leaf pigmentation (400–700 ​nm) and mesophyll structure (700–1300 ​nm). Longer wavelengths in the short-wave infrared (SWIR; 1300–2500 ​nm) range are needed to detect changes in water content [10] and biochemistry.

Machine learning algorithms can potentially help to detect subtle spectral changes in potato plants and thus support early (i.e. pre-symptomatic) detection of nematode infections and improve disease control strategies. Among the multitude of algorithms, Random Forest (RF), Support Vector Classifier (SVC), boosting algorithms (i.e. XGBoost – eXtreme Gradient Boosting; XGB) and Neural Networks (NN) are frequently used to analyze spectral data [[14], [15], [16]]. Complex models, such as deep neural networks, generally provide better predictive power and can capture complicated patterns in the data, but they require large datasets to train effectively [17].

Model transparency is key to understanding how predictions are made in order to gain meaningful insights. Methods such as SHapley Additive exPlanations (SHAP) [18] help to explain individual predictions by revealing the contribution of each feature, e.g. which spectral wavebands are most important for classification tasks. Dimensionality reduction techniques such as Uniform Manifold Approximation and Projection (UMAP) [19] complement SHAP by enabling the visualization of high-dimensional spectral data in a lower-dimensional space.

The aim of this study was to develop an explainable machine learning-based method in conjunction with hyperspectral imaging to accurately discriminate between biotic stress caused by nematode infection and abiotic stress caused by drought in potato plants. We wanted to evaluate the accuracy of discrimination between these stressors and investigate their relationships. Furthermore, we aimed to identify the key spectral bands that contribute to the model's decisions and visualize how the spectral data from different stress treatments are distributed in a low-dimensional space to gain insights into the manifestation of the stressors in the spectral signatures.

## 2 Materials and methods

### 2.1 Design and conditions of the biological experiment

The experiment was conducted in a glasshouse of the Agricultural Institute of Slovenia (Ljubljana, Slovenia) from the beginning of April to the end of June 2021. The main milestones of the experiment included the planting of potatoes on April 2, nematode inoculation on April 15, and evaluations on April 26 (Evaluation 1), May 25 (Evaluation 2), and June 25 (Evaluation 3, end of the greenhouse experiment), when height and plant physiology were measured (see Section 2.2). These evaluations coincided with the hyperspectral imaging sessions (IS – imaging session) to ensure comprehensive data collection across all experimental phases. All plants in this experiment were hyperspectrally imaged at three key time points: 10, 40 and 70 days after nematode inoculation (DAI). These time points were carefully selected to capture critical phases of plant response, including early nematode infection without visible symptoms (DAI 10), the middle of the nematode life cycle when high metabolic activity was expected (DAI 40), and the development of visible symptoms towards the end of the nematode life cycle at the beginning of cyst maturation (DAI 70). The glasshouse conditions during the experiment were natural and additional artificial 16/8-h lighting (day/night), an average temperature of 19.8 (±3.0) °C and a relative humidity of 63.2 (±12.7) %. The potato (*Solanum tuberosum*) variety Désirée was used for this experiment, as it is susceptible to *G*. *pallida* and *G. rostochiensis*. The potato tubers were planted in wide 5-L pots (26 ​cm diameter) filled with an artificial substrate mixed with 1 part coarse-grained (MP4), 3 parts fine-grained (MP1/G) quartz sand (Termit, Slovenia), with 25 ​g ​kg^−1^ kaolin (Samson Kamnik, Slovenia), 38 ​g ​kg^−1^ lightweight expanded clay aggregate Glinopor (BVB Gardening, Germany) and 5g kg^−1^ slow-release fertilizer Osmocote® Exact Standard (ICL Specialty Fertilizers, Israel), N-P-K(+Mg) ​= ​15-9-12(+2) with trace elements. Each plant was allowed to develop up to three shoots to standardize growth and any extra shoots were removed while they were still small.

**Fig. 1:**
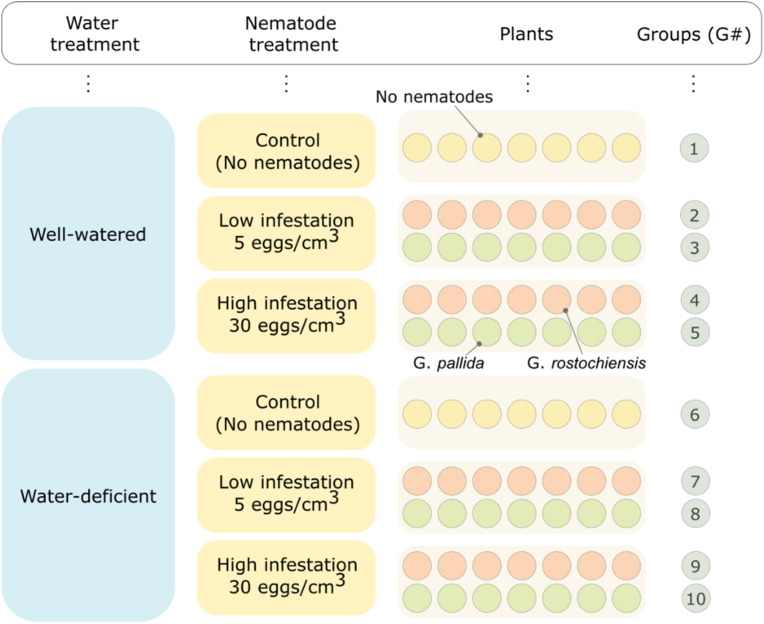
Experimental design and grouping of plants. The primary subdivision, marked in blue, separates the plants according to the amount of water they received. Within each watering group, plants were divided into three categories according to the level of nematode infestation: low inoculated, high inoculated, and non-inoculated (control). Each subgroup contained 7 plants of each nematode species, except for the control group, which contained only 7 plants as it did not contain nematodes. The final composition of the groups, labelled G# (where # ranges from 1 to 10), is used throughout the paper for annotation.

Fourteen days after planting the tubers, the potato plants were inoculated with the nematodes *G. pallida* Pa3 (Chavornay population) and *G. rostochiensis* Ro1 (Ecosse population). The nematode inocula were prepared by soaking the cysts in potato root diffusate in a glass test tube for 24h, followed by gently crushing them with a rotating metal plunger to release the eggs and second-stage juveniles (J2) contained in the cysts (modified after [20]). The nematodes in suspension (all eggs ​+ ​J2) were counted under an SMZ-800 stereomicroscope (Nikon, Japan) to prepare inoculum dilutions. Before inoculation, all plants in the experiment were watered and excess water was drained. The nematodes were introduced into the moist substrate at a depth of 10 ​cm by pipetting, evenly around the sprouting potato plants. Two inoculum levels (P_i_) were used: a low level of 5 eggs ​+ ​J2 per cubic centimeter of substrate and a high level of 30 eggs ​+ ​J2 per cubic centimeter. Two different water regimes were used: (1) a well-watered treatment in which substrate moisture was kept constant, and (2) a water-deficit treatment in which watering was stopped 3–5 days before assessment or hyperspectral imaging to distinguish between nematode infection and drought stress. The moisture content in the pots was maintained manually. The pots were weighed weekly and the moisture content was adjusted to 12–15 ​% of the dry weight of the substrate to ensure consistency between treatments. The experiment was randomized with 7 replicates per treatment, resulting in a total of 70 pots (Fig. 1). The pots were rotated weekly within each treatment and in the chamber to minimize position effects.

### 2.2 Nematode extraction, plant growth and plant physiology

The height of potato shoots was measured at 10, 40 and 70 days after inoculation (DAI) for each plant evaluation. Total leaf area, measured with the LI-3100C Area Meter (LI-COR Biosciences, USA), and plant fresh weight were measured at the end of the experiment. After the final evaluation of the plants at DAI 70, the developing cysts of *G. pallida* and *G. rostochiensis* were allowed to mature for 20 days during the subsequent drying of substrate. The mature cysts were extracted from the substrate using the modified Fenwick can method [21] and subsequently purified from plant debris by flotation in absolute ethanol. The purified nematode cysts were air-dried and counted using ImageJ image recognition software (NIH, USA). The total number of nematode cysts after the experiment (P_f_) and the initial number of cysts in the inoculum (P_i_) (assuming an average of 300 eggs per cyst) were used to calculate the nematode reproduction factor (Rf) according to the following formula: Rf = P_f_/P_i_.

General plant stress was assessed in all plant evaluations. Photosynthetic rate (Photo), stomatal conductance (gsw) and effective quantum efficiency of photosystem II (PhiPS2) were measured on the youngest fully developed, sun-exposed leaf per plant using the LI-6400XT Portable Photosynthesis System (LI-COR Biosciences, USA) at ambient air temperature (19.2 ​°C–25.5 ​°C), humidity (Rh ​= ​54.6 ​%–78.8 ​%), reference CO_2_ concentration (400 ​μmol ​mol^−1^) and stable light intensity of 1000 ​μmol photons m^−2^ ​s ^−1^ from an internal LED light source. The measurements were carried out between 9:30 and 13:00.

### 2.3 Hyperspectral data acquisition and preparation

Hyperspectral images were acquired in both the VNIR (visible to near-infrared) and SWIR (short-wave infrared) spectral ranges using pushbroom cameras Hyspex (Norsk Elektro Optikk, Oslo, Norway): VNIR-1600 (400–988 ​nm, 160 bands, 3.6 ​nm bandwidth, across-track resolution 1600 px) and SWIR-384 (950–2500 ​nm, 288 bands, 5.4 ​nm bandwidth, across-track resolution 384 px). The cameras were mounted 3 ​m above the plants in a dark room. Uniform illumination was achieved using calibrated halogen light sources, which were switched on 15 ​min before imaging to minimize thermal drift. In each imaging session, up to three potato plants were imaged next to a 20 ​% gray calibrated reflectance panel (SphereOptics, Herrsching, Germany) on a black synthetic background with less than 5 ​% reflectance.

The image pre-processing workflow consisted of six steps: (1) Radiometric calibration of the images to radiance units (*W sr*^*−1*^*m*^*−2*^) using Hyspex's proprietary software; (2) Conversion of the VNIR and SWIR images to reflectance using the reference panel located at the same height of the plants; (3) Image segmentation and extraction of leaf area pixels, separately for each independent plant; (4) Calculation of mean spectral signatures from these extracted pixels, separately for VNIR and SWIR; (5) Removing of the first and last five spectral channels from both the VNIR and SWIR, leaving a total of 428 bands; (6) Concatenating the VNIR and SWIR spectral signatures to create a single spectral signature for each potato plant. Steps (2)–(6) were performed using the open-source software SiaPy [22]. The result of this process were 70 spectral signatures, each corresponding to a single potato plant in a single imaging session. With three imaging sessions performed, the total dataset amounted to 210 spectral signatures.

### 2.4 Datasets

Several datasets were created to assess the model's ability to distinguish between data instances of different categories (C#). These categories were created based on the groups (G#) used to train machine learning models to predict discrete category variables (i.e., a classification task). Table 1 provides an overview of the datasets, each of which was assigned a unique identifier (D#). The table also indicates how the predicted variable was constructed, i.e. which groups (also identified by their own identifiers) were used in the dataset, and provides a brief description of the data instances.

**Table 1:** Summary of datasets created. Each row provides details of how a particular dataset was created and which categories or groups were used to create it. The description explains the classes used, the size of the dataset and the separations to be analyzed for each dataset. The datasets are labelled with “D#” (where # ranges from 1 to 18) and the target categories within each dataset are labelled with “C#” (where # ranges from 1 to a maximum of 4, depending on the dataset). The previously defined groups are labelled “G#“.

Dataset (D#)	Categories (C#)	Dataset size	Description
D1	C1 Є {G1} C2 Є {G6}	42	Well-watered vs. water deficient controls.
D2	C1 Є {G1, G2, G3, G4, G5} C2 Є {G6, G7, G8, G9, G10}	210	Well-watered vs. water-deficient plants, regardless of nematode inoculation.
D3	C1 Є {G4} C2 Є {G2}	42	*G. rostochiensis* high vs. low inoculation level for well-watered plants.
D4	C1 Є {G5} C2 Є {G3}	42	*G. pallida* high vs. low inoculation level for well-watered plants.
D5	C1 Є {G7} C2 Є {G9}	42	*G. rostochiensis* high vs. low inoculation level for water-deficient plants.
D6	C1 Є {G8} C2 Є {G10}	42	*G. pallida* high vs. low inoculation level for water-deficient plants.
D7	C1 Є {G3, G5} C2 Є {G2, G4}	84	*G. pallida* vs. *G. rostochiensis* for well-watered plants.
D8	C1 Є {G8, G10} C2 Є {G7, G9}	84	*G. pallida* vs. *G. rostochiensis* for water-deficient plants.
D9	C1 Є {G2, G4} C2 Є {G1}	42	*G. rostochiensis* vs. control for well-watered plants.
D10	C1 Є {G3, G5} C2 Є {G1}	42	*G. pallida* vs. control for well-watered plants.
D11	C1 Є {G7, G9} C2 Є {G6}	42	*G. rostochiensis* vs. control for water-deficient plants.
D12	C1 Є {G8, G10} C2 Є {G6}	42	*G. pallida* vs. control for water-deficient plants.
D13	C1 Є {G3, G5} C2 Є {G2, G4} C3 Є {G1}	63	*G. pallida* vs. *G. rostochiensis* vs. controls for well-watered plants.
D14	C1 Є {G8, G10} C2 Є {G7, G9} C3 Є {G6}	126	*G. pallida* vs. G*. rostochiensis* vs. controls for water-deficient plants.
D15	C1 Є {G2, G3, G4, G5} C2 Є {G1}	42	Nematode inoculation vs. control for well-watered plants.
D16	C1 Є {G7, G8, G9, G10} C2 Є {G6}	42	Nematode inoculation vs. control for water-deficient plants.
D17	C1 Є {G2, G3, G4, G5} C2 Є {G7, G8, G9, G10} C3 Є {G1} C4 Є {G6}	84	Nematode-inoculated well-watered plants vs. inoculated water-deficient plants vs. well-watered control vs. water-deficient control.
D18	C1 Є {G2, G3, G4, G5} C2 Є {G1} C3 Є {G6}	63	Nematode-inoculated well-watered plants vs. well-watered control vs. water-deficient control.

The datasets were designed to isolate independent effects corresponding to specific research questions. Dataset D1 includes only controls without nematode infection, while D2 combines controls and inoculated samples, enabling assessment of drought effects relative to nematode inoculation. Datasets D3–D6 evaluate whether different levels of nematode inoculation can be separately identified in well-watered versus water-deficient plants, revealing infection severity impacts under varying water stress conditions. Datasets D7–D8 assess the distinguishability of nematode species across water treatments. Datasets D9–D12 evaluate differentiation of individual nematode species from controls, regardless of inoculation levels, identifying species-specific effects. D13–D14 focus on modeling differentiation between nematode species and controls within each water treatment, providing insights into species-specific impacts. Datasets D15–D16 assess the overall distinction between nematode-infected and control plants, evaluating biotic stress impacts. Finally, datasets D17 and D18 examine practical applications: D17 models differentiation among controls, abiotic stress (drought), biotic stress (nematode infection), and their combination, while D18 distinguishes strictly between abiotic and biotic stresses without their interaction. Datasets D2 and D17 were analyzed in greater detail due to their key roles in capturing general watering conditions (D2) and practical differentiation among stress types (D17).

### 2.5 Processing workflow

**Fig. 2:**
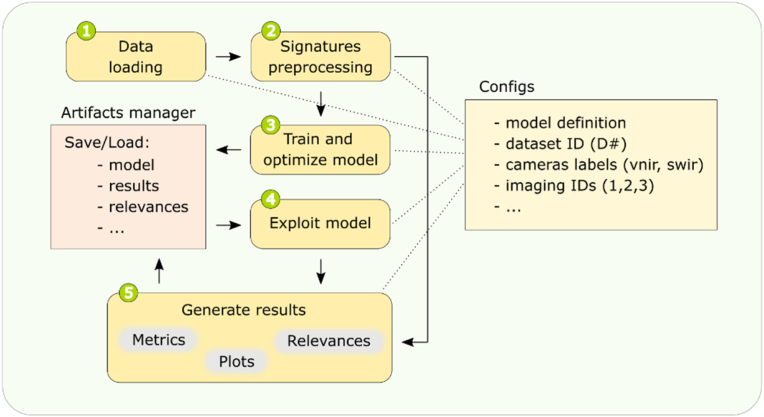
Schematic representation of the workflow. Steps 1–5 are labelled with green-circled numbers, indicating the order of processing. The arrows indicate the direction of the data flow. The “Artifact Manager” module abstracts helper functions that are responsible for the systematic saving and loading of artifacts (e.g. model checkpoints, results). The “Configs” module illustrates the global configurations that are included in each step and define important parameters such as the model selection and the choice of dataset for each run.

The processing workflow (Fig. 2) was divided into several key steps, the main objective of which was the modeling and evaluation of datasets. The pipeline, implemented in Python 3.11, ensured a systematic and organized approach to spectral data analysis. The following subsections provide a detailed breakdown of each step of the processing procedure.

#### 2.5.1 Loading of the data and pre-processing the signatures (steps 1 and 2)

Data loading was the first pipeline step, retrieving spectral signatures based on dataset ID, selected imaging sessions (typically all three sessions: 1–3), and camera data (VNIR, SWIR, or both), typically using the full VNIR ​+ ​SWIR spectrum unless otherwise specified. Loaded data were shuffled and balanced according to the classification target if one category was overrepresented. Balancing involved random subsampling of overrepresented categories to match the least represented one, ensuring equal contribution during training. The pipeline was executed multiple times with different random seeds to confirm results were independent of balancing.

Following data formatting, spectral transformations were applied. The first and last five spectral bands from both VNIR and SWIR were removed to avoid boundary noise and merged into a single signature. Signatures underwent a two-step normalization: Standard Normal Variate (SNV), correcting scattering by centering individual samples at zero mean and scaling to unit variance, and Standard Scaling (SS), standardizing across wavelengths to remove global scaling differences. Subsequently, normalized signatures underwent spectral enhancement using either Savitzky-Golay (SG) or Fast Fourier Transform (FFT) filtering to highlight subtle spectral variations and compare preprocessing methods. Optimal preprocessing hyperparameters were chosen based on performance metrics from the optimization phase (pipeline step 3).

#### 2.5.2 Training and optimizing the model (step 3)

The machine learning models were constructed by integrating a dimensionality reduction technique with a classifier that follows the specific model definition. Several models were used to obtain a comprehensive overview of the performance, taking into account that different methods may excel depending on the specific characteristics of each dataset [23]. Dimensionality reduction was applied to the spectral data to address the high dimensionality, which can lead to overfitting and increased computational cost [5]. Either Partial Least Squares (PLS) or Independent Component Analysis (ICA) were used, with the option of not using dimensionality reduction at all (no dimensionality reduction). Either Support Vector Classifier (SVC) or eXtreme Gradient Boosting (XGB) were used for classification. All possible combinations of dimensionality reduction methods and classifiers were tested. These algorithms were chosen because they represent standard machine learning approaches that are commonly used in spectral classification tasks [24,25].

Hyperparameter tuning was performed in 200 trials using the automated optimization framework, Optuna [26] and the Siapy library [22]. Optuna uses the Tree-structured Parzen Estimator (TPE) algorithm [27], a Bayesian optimization method that models the objective function to efficiently navigate the hyperparameter space. The ranges of hyperparameters for all optimized models can be found in the Supplementary Material S1. To ensure robust performance estimates, 3-times repeated 5-fold cross-validation (CV) was used [28]. The average F1 score across all categories was calculated as the optimization target, where model was trained multiple times on the training set and evaluated on the test set. To ensure reproducibility, both the model and the cross-validation were initialized with a uniform random seed in each trial. The best-performing model checkpoint (optimized hyperparameters of the model), determined by the highest F1 score, was saved for future use.

#### 2.5.3 Exploiting models and generating results (steps 4 and 5)

Checkpoints of the machine learning model could be loaded and used directly for further evaluation. To ensure consistent and unbiased results, the evaluation was performed with a 5-times repeated 5-fold cross-validation (CV), this time with a different random seed than in the optimization phase and with an increase in repetitions from 3 to 5 for additional robustness. The hyperparameters were not tuned in this step; instead, the checkpointed model configuration was evaluated as-is. Several classification metrics were calculated using CV, including F1-score, precision and recall, to provide a comprehensive assessment of the model's performance. In the case of multi-class classification, the class-specific F1 scores, precision and recall were averaged to determine the overall metrics. In addition to these metrics, various performance-related graphs and insights were generated to visually evaluate model performance and features. All metrics and visualizations were systematically stored to document data integrity, the model's decision-making process and the performance metrics achieved.

An exploratory data analysis (EDA) was performed to gain an understanding of the dataset and model behavior. The influence of individual wavelength bands on model predictions was interpreted using SHAP values [18], allowing for a more interpretable and transparent analysis of feature importance and their contribution to model results. In addition, SHAP was used to identify the most important spectral bands thus serving as a band selection algorithm. The model was then retrained and evaluated using only the selected bands to assess how the selected spectral bands affect the overall performance. To cope with the complexity of high-dimensional datasets [5], Uniform Manifold Approximation and Projection (UMAP) [19] was applied in an unsupervised manner. This dimensionality reduction technique enabled the visualization of complex relationships between the wavelength bands, which helped to identify distinct clusters and patterns that are helpful in distinguishing between groups of spectral signatures. In addition, these visualizations supported the identification of potential data patterns or anomalies that could affect model performance. Unsupervised analysis can reveal hidden patterns in the data that might be overlooked when relying solely on classification models and their results.

### 2.6 Statistical analysis

A Linear Mixed Model (LMM), fitted by Restricted Maximum Likelihood (REML), was used to analyze the effects of the different treatments on physiological parameters. The model formula used was:

$$y∼T_{\text{water}}+T_{\text{nematode}}+\left(1|\text{Date}\right)+\left(1|\text{Plant}\right)$$

where the variables are defined as follows:

- Date: An ordinal variable with the values 0, 1, and 2 representing the subsequent dates (0 ​= ​DAI 10, 1 ​= ​DAI 40, 2 ​= ​DAI 70).
- Plant: A nominal variable representing the plant ID or its identification. Tested but excluded from modeling as no variance was explained.
- $T_{\text{water}}$ (Water treatment): A categorical variable indicating irrigation conditions, either “well-watered” (ww) or “water-deficient” (wd).
- $T_{\text{nematode}}$ (Nematode treatment): A categorical variable combining the nematode type (“*G. rostochiensis*” (r), “*G. pallida*” (p) or “control” (c)) with the degree of inoculation (0 ​= ​none, 1 ​= ​low or 2 ​= ​high inoculation).

The model included fixed effects of water and nematode treatments and random intercepts for date and plant, which enabled the assessment of their influence on the response variable *y*. To stabilize variance and approximate normality, a Box-Cox transformation was applied to the data. Statistical significance for pairwise comparisons within each treatment group was assessed using p-values, with a significance threshold set at α ​= ​0.05. Differences were considered statistically significant if the p-values were below this threshold. To compare treatment effects, Analysis of Variance (ANOVA) was used to assess differences between groups. Post-hoc Tukey's Honest Significant Difference (HSD) test was used for pairwise comparisons of group means, controlling for family-specific error rate. All analyses were performed using R Statistical Software (version 4.4.1 [29]).

## 3 Results

### 3.1 Nematological parameters and plant morphology

**Fig. 3:**
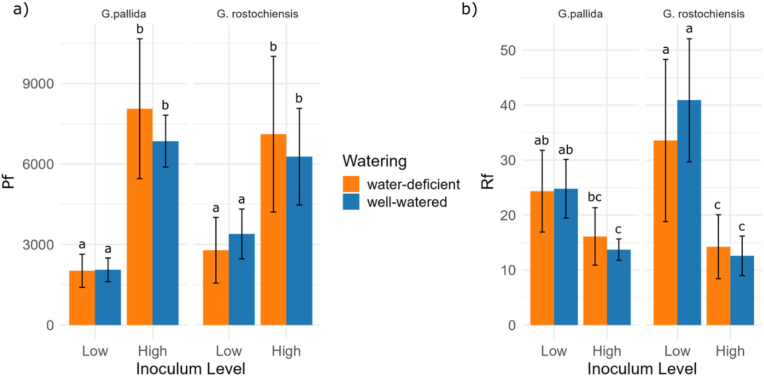
Growth parameters of the nematodes at the end of the experiment. Expressed as a) final number of mature cysts (P_f_) per plant and b) reproduction factor (Rf) where Rf = P_f_/P_i_, with P_i_ ​= ​5 or 30 eggs ​+ ​J2/cm^3^ substrate for low or high inoculum level, respectively. The columns represent the average values, the black bars indicate the standard deviation (±SD). The 4 columns on the left correspond to *G. rostochiensis*, while the 4 columns on the right represent *G. pallida*. Different letters indicate statistically significant differences between groups based on Tukey's HSD test (p ​< ​0.05). Groups sharing the same letter are not significantly different.

Both nematode species reproduced successfully at both watering regimes, reaching a mean final population of 0.5 (±0.2) cysts/cm^3^ of substrate at lower initial inoculum (P_i_) and 1.4 (±0.5) cysts/cm^3^ at higher P_i_. The final number of mature cysts and the associated reproduction factor (Rf) showed an inverse relationship influenced by the inoculum level, reaching a mean Rf of 30.9 (±12.0) at lower P_i_ and 13.7 (±5.1) at higher P_i_ (Fig. 3). There was an interaction between inoculum level and nematode species for the reproduction factor: *G. pallida* reproduced more than *G. rostochiesis* at low inoculum level, but not at high inoculum level (P ​< ​0.05) (see Supplementary Material S2 for detailed tables).

**Fig. 4:**
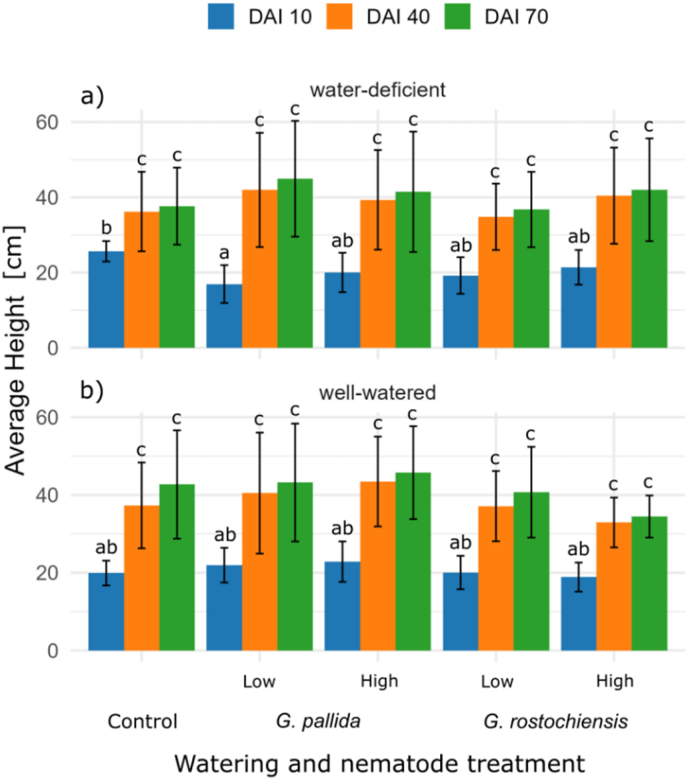
Average height of potato plants. All watering treatments: a) well-watered and b) water-deficient; and nematode treatments (*G. pallida*: low/high and *G. rostochiensis*: low/high) are shown at DAI 10, DAI 40 and DAI 70. Different letters indicate statistically significant differences between groups based on Tukey's HSD test (p ​< ​0.05). Groups sharing the same letter are not significantly different.

Morphological parameters generally showed no significant differences between treatments (see Supplementary Material S3). The only notable exception was the average height of potato plants at DAI 10, where water-deficient plants inoculated with a lower *G. pallida* inoculum were significantly shorter than the control plants. This effect was not observed in well-watered plants and no significant differences were observed later at DAI 40 or DAI 70. There were also no significant differences in leaf area and fresh plant weight between the treatment groups. The height of the potato plants increased as their phenological development stages progressed. The fastest growth was observed between DAI 10 and DAI 40, while the increase in height from DAI 40 to DAI 70 was comparatively lower (Fig. 4).

### 3.2 Unsupervised data analysis

**Fig. 5:**
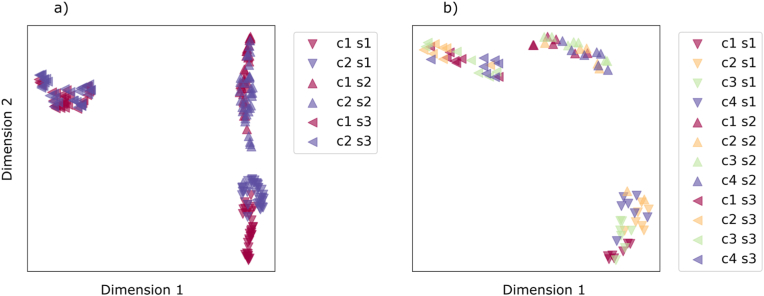
UMAP 2-dimensional data visualization. Datasets: a) D2 and b) D17. The labels S1 (DAI 10), S2 (DAI 40) and S3 (DAI 70) denote data instances acquired during consecutive imaging sessions. The annotations C# (where "#" stands for the consecutive category number) refer to the specific classification targets in Table 1.

The visualization in low-dimensional space (Fig. 5) shows that the largest deviation in spectral signatures originates from the growth stage of the plant. This is true for all datasets, as UMAP effectively splits the data instances into three different clusters that perfectly match the imaging dates and illustrate the influence of growth stage on the spectral data. However, minor differences, such as those caused by inoculation or water deficiency, are not recognisable in the 2D features generated by the method. These subtle differences only become apparent when additional dimensions are included.

### 3.3 Classification metrics

The datasets shown in Table 1 were analyzed using 12 different models to perform an initial screening for the planned classification tasks (Table 2). Due to the extensive volume of results generated by this approach, the focus here is on the F1-average score as it provides a general overview of the model performance. All classification results are presented based on the test sets generated through cross-validation.

**Table 2:** The performance achieved by each model. The cross-validated metrics were calculated for combinations of enhancement techniques (FFT or SG), dimensionality reduction methods (ICA, PLS or/– no dimensionality reduction) and classification algorithms (XGB or SVC). In all cases, the spectral signatures were normalized using SNV transformation and Standard Scaling (SS). The datasets are labelled as “D#" (where # ranges from 1 to 18). The highest F1 score achieved for each dataset is highlighted in bold text.

Enhancement	FFT						SG					
Reduction	ICA		PLS		/		ICA		PLS		/	
Classification	SVC	XGB	SVC	XGB	SVC	XGB	SVC	XGB	SVC	XGB	SVC	XGB
Dataset (D#)												
D1	0.90	0.83	0.90	0.77	0.80	0.89	0.89	0.77	**0.91**	0.78	0.80	0.82
D2	0.86	0.85	**0.95**	0.91	0.95	0.87	0.92	0.85	**0.95**	0.92	0.94	0.90
D3	**0.65**	0.54	0.61	0.52	0.53	0.63	0.56	0.57	0.61	0.58	0.52	0.61
D4	0.66	0.53	0.58	0.63	0.68	**0.74**	0.68	0.57	0.66	0.63	0.66	0.69
D5	0.67	0.66	**0.69**	0.65	0.52	0.64	0.48	0.52	0.52	0.49	0.43	0.48
D6	0.63	**0.67**	0.62	0.56	0.61	0.57	0.62	0.55	0.60	0.63	0.59	0.62
D7	0.70	0.64	0.71	0.68	0.53	0.70	0.71	0.61	**0.73**	0.69	0.65	0.71
D8	0.68	0.63	0.78	0.74	0.73	0.66	0.68	0.62	**0.80**	**0.80**	0.77	0.70
D9	**0.77**	0.60	0.70	0.68	0.69	**0.77**	0.74	0.70	0.66	0.65	0.68	0.66
D10	0.81	0.71	**0.83**	0.70	0.48	0.73	0.76	0.65	0.80	0.71	0.75	0.70
D11	0.72	0.67	0.76	0.72	0.72	**0.79**	0.77	0.69	0.67	0.60	0.73	0.66
D12	0.75	0.72	**0.80**	0.78	0.79	0.71	0.73	0.73	**0.80**	0.76	0.76	0.77
D13	0.54	0.49	0.48	0.55	0.55	**0.60**	0.53	0.49	0.52	0.49	0.55	0.50
D14	0.61	0.52	0.54	0.57	0.62	0.60	**0.63**	0.48	0.61	0.62	0.62	0.53
D15	0.68	0.61	**0.70**	0.65	0.67	0.69	0.66	0.61	0.68	0.68	**0.70**	0.63
D16	**0.80**	0.66	0.78	0.73	0.71	0.74	0.74	0.73	0.73	0.67	0.77	0.67
D17	0.52	0.45	0.52	0.50	0.54	0.56	0.52	0.48	0.52	0.48	**0.58**	0.53
D18	0.62	0.54	**0.64**	**0.64**	0.55	0.60	0.63	0.56	**0.64**	0.60	0.63	0.62

The results show that water deficit stress (abiotic stress) is the most important factor for the differentiation between the target categories. This is evident both in the classification of control plants (D1), where a maximum F1 value of 0.91 is achieved and in the inoculated plants (D2), which achieve a maximum value of 0.95. These results indicate that drought stress exerts a more dominant influence on classification results than nematode inoculation. Differentiation between inoculation levels (low vs. high), when focusing on a single nematode species and one water treatment at a time (D3–D6), yields moderate F1 between 0.65 and 0.74, with *G. pallida* achieving the highest F1 in this range. In contrast, when differentiating between nematode species (D7–D8), a higher average F1 value per water treatment was obtained when both inoculation levels were considered simultaneously. This approach resulted in maximum accuracies of 0.73 and 0.80 for well-watered and water-deficient plants, respectively. In addition, distinguishing a single nematode species from the control within the same water treatment (D9–D12) resulted in consistently high F1 values, ranging from 0.77 to 0.83, demonstrating a comparatively high classification performance. However, classification between nematode species and control plants at the same time (D13–D14) showed relatively low performance, with F1 ranging from 0.60 for well-watered plants to 0.63 for water-deficient plants. Infection detection (D15) reached a maximum F1 of 0.70. In contrast, discriminating between potato plants exposed to both biotic and abiotic stress and those exposed to abiotic stress only (D16) resulted in a higher F1 of 0.80. When all categories from D15 and D16 were combined into a single classification task (D17), the F1 decreased to 0.58. This decrease is due to the increased complexity of discriminating between four classes simultaneously, as opposed to the binary classifications in D15 and D16. When healthy plants are compared exclusively to either biotically or abiotically stressed plants (D18), but not both simultaneously, the model achieves a relatively low F1 of 0.64. Datasets D2 and D17 represent the opposite ends of the performance spectrum—D2 scored the highest, while D17 scored the lowest.

**Fig. 6:**
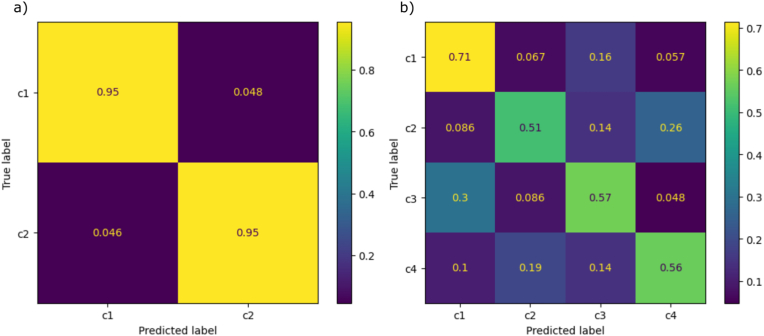
Confusion matrices for classifications using datasets D2 and D17. Illustration of the model performance for the datasets a) D2 and b) D17, evaluated with the FFT-PLS-SVC and SG-SVC models. Lighter colors represent higher accuracy scores, with a corresponding bar chart for reference. The annotations C# (where "#" stands for the consecutive category number) refer to the specific classification targets in Table 1.

The datasets D2 and D17 were further analyzed by category (Fig. 6) to evaluate the performance of the top-performing models more thoroughly: FFT-PLS-SVC and SG-SVC, fitted to the data. For D2, accuracies for both classes reached 0.95, indicating a high degree of differentiation between well-watered and water-deficient plants. In contrast, D17 showed greater variability in performance. The highest accuracy of 0.71 was achieved for the inoculated, well-watered category (c1), while the inoculated, water-deficient category (c2) showed the lowest performance. The model had the most difficulty with categories c1 and c3 (well-watered plants), with c3 being misclassified as c1 in 30 ​% of cases, and with c2 and c4 (water-deficient plants), with c2 being misclassified as c4 in 26 ​% of cases. These misclassifications indicate that water treatment is a challenge for the model.

The performance of the best models for datasets D2 and D17 was evaluated using F1 score, precision and recall, with results reported for individual imaging sessions (IS) and for the pooled data (Table 3). For the D2 dataset, the FFT-PLS-SVC model showed consistently high performance for all IS and pooled data. F1 scores ranged from 0.94 (±0.07) in IS 1 to 0.96 (±0.06) in IS 3, with a pooled F1 value of 0.95 (±0.04). Precision and recall were also robust, with pooled values of 0.96 (±0.04) and 0.95 (±0.04), respectively. In contrast, the SG-SVC model for dataset D17 showed more variable and lower performance. F1 values ranged from 0.47 (±0.22) in IS 3 to 0.55 (±0.20) in IS 1, with a pooled F1 value of 0.58 (±0.13). Precision and recall followed a similar trend, with pooled values of 0.63 (±0.14) and 0.59 (±0.12), respectively.

**Table 3:** Performance of the best model (FFT-PLS-SVC) for dataset D2 and model SG-SVC for dataset D17. F1 value, precision and recall are reported separately for each imaging session (IS) and for the combined data (pooled). The results are given as mean values with standard deviations (±SD).

Dataset	IS	F1	Precision	Recall
D2	1	0.94 (±0.07)	0.95 (±0.07)	0.95 (±0.06)
	2	0.95 (±0.09)	0.97 (±0.05)	0.96 (±0.09)
	3	0.96 (±0.06)	0.96 (±0.05)	0.96 (±0.06)
	Pooled	0.95 (±0.04)	0.96 (±0.04)	0.95 (±0.04)
D17	1	0.55 (±0.20)	0.56 (±0.23)	0.60 (±0.19)
	2	0.53 (±0.28)	0.53 (±0.29)	0.60 (±0.26)
	3	0.47 (±0.22)	0.49 (±0.24)	0.52 (±0.22)
	Pooled	0.58 (±0.13)	0.63 (±0.14)	0.59 (±0.12)

### 3.4 Statistical analysis of the physiological parameters

Linear mixed models showed that the explanatory variables r.1 and wd significantly affected Photo and PhiPS2, while only wd significantly influenced gsw. Estimates for r.1 were positive, indicating increased values associated with higher r.1, whereas wd had negative estimates, indicating decreased values under water deficiency. Other explanatory variables had positive but non-significant estimates, indistinguishable from random variation (Supplementary material S4).

Random effects analysis revealed substantial unexplained variance. Among random factors, Plant ID did not explain any variation, while Date contributed minimally to overall variance (Supplementary material S5–S6). Residual variance significantly exceeded the variance explained by Date, ranging from 1.8-times higher (PhiPS2) to 13-times higher (Photo), suggesting most variability was due to unmeasured factors beyond fixed effects or random grouping.

Physiological parameters varied distinctly across treatments and imaging sessions (IS)Supplementary Material S7. Photo was consistently higher in well-watered conditions, especially during IS3, compared to water-deficient treatments, which showed greater variability. Similar trends occurred in gsw, with higher values under well-watered conditions (particularly treatments p.1 ww and p.2 ww) during early stages (IS1), while water-deficient controls (c.0) consistently had the lowest gsw values across all stages. Generally, gsw values declined over time (IS1 ​> ​IS3). PhiPS2 exhibited minimal variability but higher values under well-watered conditions, with the lowest values generally occurring at IS2 across most treatments, except r.2 wd. Control treatments consistently had the lowest physiological values, highlighting the negative impacts of nematode infection and water deficiency.

**Fig. 7:**
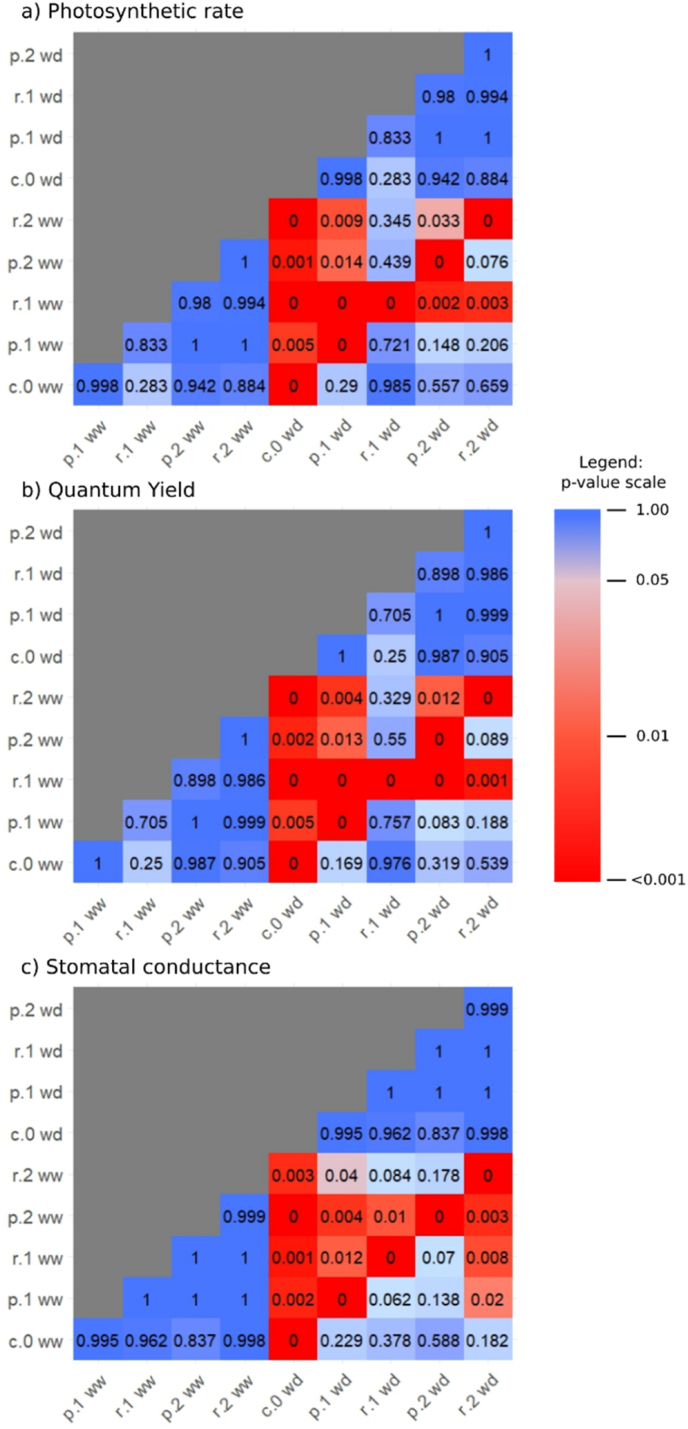
Pairwise comparisons of plant physiological variables in all treatment groups. Given are p-values for each pair of all treatment groups. Detailed results are provided in supplementary S4-S6. a) photosynthesis rate, b) quantum yield and c) stomatal conductance. Watering conditions are labelled as “well-watered” (ww) or “water-deficient” (wd). Nematode treatments are labelled with the species - “*G. rostochiensis*” (r), “*G. pallida*” (p), or “control” (c) - and inoculation levels: 0 (none), 1 (low) or 2 (high). Significant differences between pairs are highlighted in red, while non-significant differences are shown in blue. The intensity of the red color shading corresponds to the p-value: lighter shading represents values close to the significance threshold (α ​= ​0.05), while darker shading indicates more extreme p-values (closer to 0). An accompanying color scale provides a visual reference for the color shades and their corresponding p-values.

P-value analyses (Fig. 7) showed no significant pairwise differences within watering groups, irrespective of nematode species or inoculation level. Significant differences emerged primarily between well-watered and water-deficient treatments, particularly comparing water-deficient controls against biotic stress treatments under well-watered conditions. Comparisons of identical treatments under contrasting watering conditions consistently yielded significant differences. Photosynthetic rate and quantum yield shared similar p-value patterns, while stomatal conductance showed slight variations. Low inoculation with *G. rostochiensis* under well-watered conditions differed significantly from other treatments for Photo and PhiPS2, while high inoculation differed from all except low inoculation under well-watered conditions. Similarly, low inoculation with *G. pallida* under water-deficient conditions differed from most treatments, except the well-watered control. For stomatal conductance, the most prominent significant difference was high inoculation with *G. pallida* under well-watered conditions, followed by high inoculation with *G. rostochiensis* under water-deficient conditions, differing from all treatments except controls.

### 3.5 The most informative spectral bands

In dataset D2, the most important regions included 750–850 ​nm, 1450–1750 ​nm (a broad range with some less important sections), and 2150–2350 ​nm (Supplementary material S8). In addition, some narrower regions, such as around 1150 ​nm, were identified as important. For the D17 dataset, the 1600–1750 ​nm range was similarly important. However, the relevant regions in D17 appeared broader and more contiguous compared to the relatively scattered regions in D2. In particular, the VNIR ranges around 440 ​nm and 630 ​nm were more influential in D17 than in D2. In contrast, a narrow band around 1050 ​nm was important for D2, but not for D17. These observations emphasize both the different spectral relevance of the individual dataset and the overlapping regions that are important for both.

### 3.6 Performance on reduced spectral bands

The performance metrics – F1 score, precision and recall – improve with increasing number of spectral bands for both datasets (Supplementary material S9), indicating better performance when more spectral information is included. They show closely aligned trends and generally increase together as the number of selected spectral bands increases. For dataset D2, all three metrics start at comparable levels and grow steadily as additional spectral bands are added. Even though the growth rates vary slightly at certain points, precision is consistently ahead of the other metrics with a lower number of spectral bands. The metrics experience a steep increase and stabilize at high values, with F1 reaching approximately 0.90 after about 150 spectral bands. For dataset D17, the metrics also follow a similar upward trend, but show stronger fluctuations with fewer spectral bands. Precision tends to lead recall and F1 score across much of the F1 score, although the differences gradually decrease as the number of spectral bands increases. Beyond 150 spectral bands, the metrics converge, with all three approaching a value of 0.60 ​at the maximum band count. Overall, the metrics in both datasets are closely related, with precision generally slightly ahead, followed by recall and F1 score.

## 4 Discussion

The classification performance varied across datasets, with the best-performing models achieving accuracy scores ranging from 0.60 to 0.95. Notably, differentiating between water treatments using machine learning proved significantly easier than identifying nematode inoculation. Similarly, analyses of physiological parameters consistently showed no statistically significant differences in pairwise comparisons within the same watering group, regardless of species or the level of nematode inoculation. This result was consistent even though the nematodes G. *pallida* and G. *rostochiensis* were successfully propagated on potato plants under both watering regimes.

The potato plants were successfully infected with nematodes, as indicated by the achieved mean population densities. At a low initial inoculum (Pi), the mean final population density ($\bar{P_{f}}$) reached 154 (±60) eggs/cm^3^ substrate, while at high Pi, $\bar{P_{f}}$ increased to 412 (±153) eggs/cm^3^ substrate, assuming an average of 300 eggs per cyst. The final nematode densities showed no significant differences between the two species. The high Pi value was well above the threshold value of 20 eggs (g soil)^−1^, which is considered likely to cause economic damage in potato [30]. However, the parameters of plant morphology (plant height, leaf area and plant fresh weight) were generally not affected by the high nematode proliferation in the trials, compared to the control (Supplementary material S5). This is evidenced by the absence of visible aboveground symptoms on the affected potato plants, which was previously reported despite an established PCN infection [6].

Despite the high initial PCN inoculum levels applied (5 and 30 eggs/cm^3^), no significant differences in plant height, leaf area, or fresh weight were observed between control and infested plants grown under greenhouse conditions. Previous studies have shown that under optimal growth conditions, potatoes can endure much higher PCN densities without growth loss than in the field [31,32]. A susceptible potato cultivar showed no yield reduction below ∼35 eggs/g soil in a controlled environment, whereas in field conditions even ∼2 eggs/g caused significant yield suppression [33]. In field environments, susceptible cultivars may exhibit clear foliar symptoms such as stunting, yell owing, or wilting under high PCN pressure due to compounded stress from suboptimal soil, variable moisture, and nutrient competition. Symptoms can also be subjected to seasonal variation and can be less severe despite higher nematode pressure [34]. Haverkort et al. [35] further showed that effects of drought and cyst nematode infection on plant growth and water relations were not always additive and nematode infection may influence effects of drought on plants. In controlled conditions with no additional limitations, even susceptible genotypes can partially compensate for root damage through enhanced resource availability and reduced transpiration demand. This underscores how greenhouse conditions can mask biotic stress effects, raising the threshold at which PCN causes visible damage. Even at our high inoculum levels, the lack of aboveground symptoms suggests that root damage was largely compensated by the plant. While this absence of visible stress limits the sensitivity of phenotypic observations (i.e. making it harder to detect infection through growth parameters alone), it reinforces the need to assess early or subclinical indicators of PCN stress, such as subtle biochemical changes, which our results show can be detected using hyperspectral imaging.

Statistical analysis indicated water stress as the primary influencing factor for physiological parameters, with significant negative relationships between water deficiency (wd) and both stomatal conductance (gsw) and photosystem II efficiency (PhiPS2), highlighting plant sensitivity to drought (Fig. 7). The decline in PhiPS2 under drought reflects impaired photochemical processes, typically resulting from damaged photosynthetic apparatus and reduced electron transport [36]. Reduced gsw under water-deficient conditions is a common plant adaptation to minimize transpiration and water loss, concurrently limiting CO_2_ uptake and photosynthetic rates [37]. The observed decline in the rate of photosynthesis under drought stress integrates the effects of reduced stomatal conductance and impaired photochemical efficiency on carbon assimilation and growth [38]. Low-level infection with *G. rostochiensis* (r.1) under well-watered conditions positively impacted photosynthetic performance and photosystem II efficiency, suggesting a potential hormetic effect where mild biotic stress enhances physiological activity. However, other nematode inoculation levels (r.2, p.1, p.2) and combinations with drought stress did not significantly affect physiological parameters under tested conditions. Infected plants could not be distinguished from non-inoculated controls based solely on these parameters, likely due to overlapping symptoms of biotic and abiotic stresses. Such masking of nematode infection symptoms by drought stress aligns with previous studies, underscoring the complexity and cumulative impact of interacting stressors on plant physiology [6,39].

Analysis of random effects revealed substantial unexplained variance. Among the included random factors, Date contributed moderately, suggesting temporal influences such as phenological development affected physiological responses. Plant ID did not explain any variation, indicating minimal differences among individual plants. The dominance of residual variance (ranging from 1.8 to 13 times greater than Date) highlights the complexity of physiological responses and suggests significant roles for additional unmeasured biotic or abiotic factors in shaping the observed variability.

The presented machine learning modeling approach demonstrated its effectiveness in isolating and analyzing the different effects of various stressors on potato plants. This was especially crucial as the most pronounced variations in spectral signatures were observed across the three imaging sessions (IS1, IS2, and IS3; Fig. 5). This was probably due to the different phenological stages of the plants in the different imaging sessions and the evolving effects of biotic and abiotic stress factors. While at IS2, nematode parasitism was expected to induce the most prominent stress signal in the plants due to the high metabolic rate of developing nematodes, IS3, however, coincided with the rapid development of potato tubers, which are a significant nutrient sink [40], which probably influenced the spectral signatures in IS3. Spectral changes were more pronounced, compared to physiological measurements, because hyperspectral imaging provides a comprehensive view of plant condition, capturing both biochemical and structural alterations over time [41]. This underscores the advantage of spectral data in detecting plant stress dynamics before they become physiologically limiting.

The identification of water stress was very successful as an almost perfect differentiation was achieved (F1 of 0.95 ​%), whereas differentiating between biotic and abiotic stress types proved more challenging. Here the model performed poorly and achieved F1 scores of less than 70 ​%, in some cases even less than below 60 ​%. It was previously demonstrated that nematode infection and drought stress elicited fundamentally similar biochemical and physiological responses in potato plants, since both led to increased abscisic acid (ABA) levels in leaves, which further increased with concurrent biotic and abiotic stresses on the same plants [42]. Changes in ABA levels significantly influence physiological responses and thus water movement in plants [43]. This could explain water stress being more easily distinguishable than nematode-induced biotic stress, as well as more difficult differentiation between the two stresses. Similar results were previously obtained in hyperspectral imaging of tomato [44].

Differentiating nematode inoculation levels proved difficult, with a maximum F1 score of 0.74, even when watering conditions were controlled. This indicates that hyperspectral signatures associated with nematode stress may not exhibit a strong, consistent gradient across different infestation levels, possibly due to plant compensatory mechanisms, overlapping stress responses, or the patchy distribution of nematode-induced damage [44]. Classification between nematode species and control plants showed F1 scores ranging from 0.60 to 0.63, highlighting significant overlap of spectral signatures of different nematodes and consequent limited differentiation. Due to the biology of nematode-plant parasitism, plants' responses to the tested nematodes likely appear similar, at least in terms of their spectral signatures [6,45,46]. The models also performed poorly (F1 score of 0.58) when tasked with classifying four classes simultaneously (biotic, abiotic, combined biotic and abiotic, and healthy plants). Simplifying the task to three classes (excluding combined stress) improved performance by a small margin (F1 of 0.64). Binary classification problems under controlled conditions (stress effect isolated) yielded better results, indicating sufficiently distinct patterns from nematode-induced stress. However, when the task involved multivariate classifications (multiple stress conditions and their combinations) the performance decreased. This aligns with the well-established principle in machine learning that classification performance often declines as the number of classes increases. The addition of more classes inherently introduces greater complexity and potential overlap between categories, making it more challenging for the model to accurately distinguish between them [47,48].

SHAP-derived relevance scores identified and ranked features by their contribution to the modeling capability, resulting in progressively enhanced classification performance as more features were integrated. Strong performance is possible with just a few wavelengths when making predictions on tubers [13,49]. However, for datasets D2 and D17, information was found to be spread across numerous wavelengths, with performance only stabilizing after around 150 bands were included. This aligns with the findings of Lapajne et al. [50] and Cui et al. [51], who similarly demonstrated the distribution of relevant spectral bands across a broader wavelength spectrum, when predicting water-deficiency in potato plants. The number of spectral bands required for classification typically increases with task complexity, as challenging problems with subtle class distinctions demand a richer set of features to achieve accurate separation [50,52]. Whilst wavelengths identified as relevant are often suitable for the development of cost-effective multispectral cameras [53], this approach proves impractical for the datasets tested in our study.

A comparison between datasets D2 and D17 revealed notable differences in the spectral bands that most effectively distinguished between groups (Supplementary material S8). Dataset D2 displayed more numerous, narrower influential bands across the spectrum compared to D17. Particularly influential in D2 were bands around 764–767, 1047, and 1450–1493 ​nm, associated with O-H groups in alcohols, phenols, and starch, as well as bands near 770, 1065, 1200, and 1453 ​nm linked to O-H bonds in water molecules. These water-related spectral regions strongly influenced D2 due to differences in water availability among plants. In the SWIR region, bands around 1613–1655, 1703–1733, 2188–2220, 2270–2347, and 2407 ​nm, associated with C-H bonds in starch, lignin, hydrocarbons (including lipids, oils, and waxes), proteins (N-H bonds), cellulose (O-H/C-H), and sugars (O-H/C-O), also impacted classification in D2 [54] The most obvious were the spectra associated with water content, which were not categorized as influential in dataset D17. [55]. Similarly, plant phenolic compounds are secondary metabolites generally derived [56,57]. Spectral signatures associated with C-H bonds in aliphatic and aromatic hydrocarbons include aliphatic glucosinolates [[58], [59], [60]] Spectral bands around 950–1490 and 1800–2500 ​nm were significant only for D2, while visible spectrum bands (around 440 and 630 ​nm) linked to carotenoids and chlorophylls, respectively, were important for D17. These were associated with leaf pigments, i.e. [61,62]. Overlapping influential spectral regions for both datasets (around 800–915, 1500, and 1600–1735 ​nm) relate to O-H groups, hydrocarbons, and protein N-H bonds, highlighting biochemical responses common to nematode parasitism and water stress [54].

This study highlights the potential of hyperspectral imaging combined with machine learning to monitor stress factors in potato plants, providing valuable insights into the application of modern technological solutions for advancing agricultural practices. The primary objective was to investigate classification task difficulties, emphasizing understanding over model complexity or optimization. The collected data serves as a valuable resource for related research, particularly in water management and plant health monitoring, addressing the increasing prevalence of soil-borne pests associated with climate change [63]. However, some important considerations need to be made. The models in this study were constructed based solely on spectral features, excluding potentially valuable data such as spatial texture information [64,65]. The use of complementary data sources, e.g. spatial information from point clouds or LiDAR, could improve model performance [66]. Dataset size, limited potato varieties, and nematode species may affect model accuracy and generalizability. Practical constraints limited dataset size, making estimates less stable and more susceptible to variability and overfitting; repeated cross-validation improves robustness but does not fully overcome this limitation [67]. Another potential limitation is the use of gravimetric estimation of watering status, which leads to uncertainties regarding the actual water deficit of plants [68]. Direct soil moisture measurements, e.g. using tensiometers, would provide more accurate and timely data.

Future research should focus on conducting field-based studies to validate and refine the models in real-world agricultural settings, ensuring their applicability extends beyond controlled laboratory environments. Additionally, interactive effects between biotic and abiotic stresses, as well as the mechanistic underpinnings of the observed responses, should be studied in more detail. Such studies would address the inherent complexities and variability of actual field conditions, significantly enhancing the robustness and reliability of the findings. Expanding the dataset to encompass a broader range of potato varieties, nematode species, and environmental conditions is critical for improving the generalizability and predictive performance of the models. Moreover, incorporating independent datasets for validation would further strengthen the reliability of the models and enhance their applicability beyond the specific conditions of this study. In addition, integrating complementary data sources could provide a more comprehensive understanding of plant stress responses, thereby elevating classification accuracy and practical utility.

## 5 Conclusions

This study investigated early detection of potato cyst nematodes using a hyperspectral VNIR-SWIR dual-sensor system. Through the machine learning approach, various datasets were analyzed to assess how stress conditions in potato plants affect the performance of machine learning models in distinguishing stress types. The results demonstrate that while technology shows high discrimination accuracy in some scenarios, challenges remain that highlight the current limitations and potential applications of hyperspectral imaging in agriculture. The spectral channel analysis further identified key input channels that provide the most valuable information for stress detection. The findings underline the need for additional data collection and more extensive analyses to validate the models’ performance and improve their robustness. Expanding the study to field conditions would enhance its practical relevance and facilitate the integration of hyperspectral imaging into precision agriculture. These advancements could contribute to more effective stress monitoring, promoting sustainable farming practices and addressing the challenges posed by a changing agricultural landscape.

## Author contributions

J. L.: Conceptualization, Methodology, Software, Formal analysis, Investigation, Data Curation, Writing - Original Draft, Writing - Review & Editing, Visualization. N. S.: Conceptualization, Methodology, Investigation, Formal analysis, Data Curation, Writing - Original Draft, Writing - Review & Editing. A. V.: Methodology, Investigation, Resources, Formal analysis, Writing - Original Draft. B. G. S.: Writing - Review & Editing. N. V.: Conceptualization, Methodology, Writing - Review & Editing. J. B.: Conceptualization, Methodology, Writing - Review & Editing. D. N.: Conceptualization, Methodology, Writing - Review & Editing. S. Š.: Conceptualization, Resources, Supervision, Project administration, Writing - Review & Editing. U. Ž.: Conceptualization, Resources, Methodology, Validation, Writing - Review & Editing, Supervision, Project administration, Funding acquisition.

## Code and data availability

We have ensured that the data and code are easily available. Researchers can access processed hyperspectral data, morphology and physiology measurements on https://zenodo.org/records/14267877 (accessed on October 5, 2025) and the code repository at https://github.com/Manuscripts-code/Potato-plants-nemdetect--PP-2025 (accessed on October 5, 2025). The segmentation, extraction of spectral signatures, and the entire optimization process were facilitated by the SiaPy Python library, available at: https://github.com/siapy/siapy-lib (accessed on February 20, 2025). We provide open access to these resources to foster collaboration, replication, validation, and further exploration of our findings.

## Funding

The research was supported by funds from the European Food Safety Authority (grant GP/EFSA/ALPHA/2018/02), and the Slovenian Research and Innovation Agency (ARIS) (MR 54720, P4-0072, P4-0431). Some of the research was conducted using equipment financed by EU-FP7 project CropSustain (FP7-REGPOT-CT2012-316205), and research infrastructure ELIXIR-SI (https://elixir-slovenia.org), funded by the European Regional Development Fund, the Slovenian Ministry of Education, Science and Sports, and by the Slovenian Research and Innovation Agency. The position and opinions presented in this paper are those of the authors alone and do not necessarily represent the views of EFSA.

## Declaration of competing interest

The authors declare that they have no known competing financial interests or personal relationships that could have appeared to influence the work reported in this paper.
