# Supplementary material for: Detecting nematodes in potato plants an explainable machine learning approach for detection of potato cyst nematode infections using hyperspectral imaging
Source: Plant Phenomics. 2025 Oct 14;7(4):100127. doi: 10.1016/j.plaphe.2025.100127 (PMC13109338; doi:10.1016/j.plaphe.2025.100127)

Supplementary Materials

**S1**: Hyperparameters of algorithms explored in optimization procedure.

| Method | Parameter | Range/Choices |
| --- | --- | --- |
| SG | win_length | 3 to 15 |
| FFT | shape_param | 0.1 to 2.0 |
|  | sigma | 0.1 to 5.0 |
| PLS | n_components | 3 to 20 |
| ICA | n_components | 3 to 20 |
|  | max_iter | 200 to 1000 |
|  | tol | 1e-4 to 1e-1 (log) |
|  | algorithm | parallel, deflation |
|  | fun | logcosh, exp, cube |
| XGB | n_estimators | 100 to 1000 |
|  | max_depth | 3 to 10 |
|  | learning_rate | 1e-3 to 10 (log) |
|  | min_child_weight | 0.1 to 10.0 (log) |
|  | subsample | 0.5 to 1.0 |
|  | colsample_bytree | 0.5 to 1.0 |
|  | reg_lambda | 1.0 to 10.0 (log) |
|  | gamma | 1e-3 to 5.0 (log) |
|  | reg_alpha | 1e-3 to 5.0 (log) |
| SVC | C | 0.1 to 10000 (log) |
|  | gamma | 1e-7 to 1 (log) |

**S2**: Variables affecting the estimates of the final number of mature cysts () and reproduction factor (Rf). The effects of Watering, Nematode species, and Inoculum levels (plus their interactions) on the target variable are examined. Watering conditions are classified as "well-watered" (ww) or "water-deficient" (wd). Nematode treatments include "G. *rostochiensis*" (r), "G. *pallida*" (p) or "control" (c), with inoculation levels of 0 (none), 1 (low) or 2 (high). Statistical significance is indicated according to the following conventions: *** for p < 0.001, ** for p < 0.01, * for p < 0.05, and one dot (.) for p < 0.1. Data are presented as mean values with standard deviation (±SD), ANOVA statistics (F-statistic with p-value) and Tukey’s HSD results. Mean values with a common letter (e.g. a, b or ab) are not significantly different at p < 0.05. The target variable was log-transformed to normalize the data distribution and meet the assumptions of the statistical analysis.

| Treatment | Number of mature cysts (P_f_) | Reproduction factor (Rf) | Significance |
| --- | --- | --- | --- |
| p.1 wd | 2021.0 ± 617.2 a | 24.3 ± 7.4 ab |  |
| p.2 wd | 8062.7 ± 2608.5 b | 16.1 ± 5.2 bc |  |
| r.1 wd | 2785.4 ± 1224.2 a | 33.6 ± 14.8 a |  |
| r.2 wd | 7112.3 ± 2900.5 b | 14.2 ± 5.8 c |  |
| c.0 wd | 0 ± 0 | 0 ± 0 |  |
| p.1 ww | 2056.1 ± 441.8 a | 24.8 ± 5.3 ab |  |
| p.2 ww | 6852.1 ± 968.5 b | 13.7 ± 1.9 c |  |
| r.1 ww | 3394.1 ± 927.4 a | 40.9 ± 11.2 a |  |
| r.2 ww | 6274.9 ± 1803.1 b | 12.6 ± 3.6 c |  |
| c.0 ww | 0 ± 0 | 0 ± 0 |  |
| ANOVA statistics |  |  |  |
| Watering | F = 0.04, p = 0.847 | F = 0.04, p = 0.833 |  |
| Nematode | F = 2.06, p = 0.158 | F = 2.08, p = 0.156 |  |
| Inoculum | F = 153.93, p = 0.000 | F = 80.87, p = 0.000 | *** |
| Watering — Nematode | F = 0.59, p = 0.445 | F = 0.59, p = 0.446 |  |
| Watering — Inoculum | F = 2.25, p = 0.140 | F = 2.22, p = 0.143 |  |
| Nematode — Inoculum | F = 9.37, p = 0.004 | F = 9.29, p = 0.004 | ** |
| Watering — Nematode — Inoculum | F = 0.26, p = 0.612 | F = 0.25, p = 0.619 |  |

**S3**: Morphological characteristics of plants in different treatments. Plant height was recorded at each imaging session (IS 1-3), while leaf area and fresh weight were recorded at the end of the experiment when the plants were cut off. Data are presented as mean values with standard deviation (±SD), ANOVA statistics (F-statistic with p-value) and Tukey’s HSD results. Mean values with a common letter (i.e. a, b or ab) are not significantly different at p < 0.05. Irrigation conditions are labeled as "well-watered" (ww) or "water-deficient" (wd). Nematode treatments are labeled with the species - "*G. rostochiensis*" (r), "*G. pallida*" (p), or "control" (c) - and inoculation levels: 0 (none), 1 (low), or 2 (high). The target variable was log-transformed to normalize the data distribution and meet the assumptions of the statistical analysis.

| Treatment | Plant height IS1 (cm) | Plant height IS2 (cm) | Plant height IS3 (cm) | Leaf area (cm^2^) | Plant fresh weight (g) |
| --- | --- | --- | --- | --- | --- |
| p.1 ww | 22.0 ± 4.5 ab | 40.5 ± 15.6 | 43.2 ± 15.2 | 1016.2 ± 148.3 | 66.6 ± 15.4 |
| p.2 ww | 22.9 ± 5.2 ab | 43.5 ± 11.6 | 45.7 ± 12.0 | 796.9 ± 97.4 | 55.5 ± 6.4 |
| r.1 ww | 20.1 ± 4.3 ab | 37.1 ± 9.1 | 40.7 ± 11.7 | 893.1 ± 70.2 | 56.9 ± 5.2 |
| r.2 ww | 18.9 ± 3.7 ab | 32.9 ± 6.4 | 34.5 ± 5.4 | 873.0 ± 108.6 | 59.1 ± 7.0 |
| c.0 ww | 19.9 ± 3.2 ab | 37.3 ± 11.0 | 42.7 ± 14.0 | 810.6 ± 139.1 | 51.9 ± 9.4 |
| p.1 wd | 16.9 ± 5.0 a | 42.0 ± 15.2 | 44.9 ± 15.4 | 982.8 ± 295.7 | 63.5 ± 24.0 |
| p.2 wd | 20.0 ± 5.2 ab | 39.3 ± 13.2 | 41.4 ± 16.0 | 912.5 ± 204.0 | 65.9 ± 17.0 |
| r.1 wd | 19.2 ± 4.9 ab | 34.8 ± 8.8 | 36.8 ± 10.0 | 835.5 ± 249.2 | 64.0 ± 19.5 |
| r.2 wd | 21.4 ± 4.6 ab | 40.4 ± 12.8 | 42.0 ± 13.7 | 878.9 ± 143.0 | 61.3 ± 14.4 |
| c.0 wd | 25.6 ± 2.7 b | 36.2 ± 10.6 | 37.6 ± 10.2 | 760.2 ± 100.8 | 51.5 ± 5.4 |
| ANOVA statistics | F=2.09, **p=0.045** | F=0.55, p=0.830 | F=0.57, p=0.814 | F=1.57, p=0.146 | F=1.11, p=0.368 |

**S4**: Estimates of the LLM with fixed effect for the physiology parameters. The estimates include estimated coefficients (Estimate), standard errors (Std. Error), t-values (t value), and p-values (Pr(>|t|)). Statistical significance is indicated according to the following conventions: *** for p < 0.001, ** for p < 0.01, * for p < 0.05, and a dot (.) for p < 0.1.

| Parameter | Variables | Estimate | Std. Error | t.value | Pr(>\|t\|) | Significance |
| --- | --- | --- | --- | --- | --- | --- |
| Photo | (Intercept) | 0,128 | 0,210 | 0,607 | 0,565 |  |
|  | p.1 | 0,168 | 0,196 | 0,857 | 0,392 |  |
|  | r.1 | 0,488 | 0,196 | 2,485 | 0,014 | * |
|  | p.2 | 0,264 | 0,196 | 1,343 | 0,180 |  |
|  | r.2 | 0,298 | 0,196 | 1,516 | 0,131 |  |
|  | wd | -0,743 | 0,124 | -5,980 | 9,97E-09 | *** |
| gsw | (Intercept) | 0,202 | 0,283 | 0,715 | 0,522 |  |
|  | p.1 | 0,177 | 0,188 | 0,943 | 0,347 |  |
|  | r.1 | 0,236 | 0,188 | 1,257 | 0,210 |  |
|  | p.2 | 0,305 | 0,188 | 1,620 | 0,107 |  |
|  | r.2 | 0,153 | 0,188 | 0,815 | 0,416 |  |
|  | wd | -0,755 | 0,119 | -6,337 | 1,5E-09 | *** |
| PhiPS2 | (Intercept) | 0,141 | 0,364 | 0,390 | 0,726 |  |
|  | p.1 | 0,123 | 0,175 | 0,703 | 0,482 |  |
|  | r.1 | 0,446 | 0,175 | 2,549 | 0,012 | * |
|  | p.2 | 0,186 | 0,175 | 1,067 | 0,287 |  |
|  | r.2 | 0,256 | 0,175 | 1,462 | 0,145 |  |
|  | wd | -0,688 | 0,110 | -6,217 | 2,86E-09 | *** |

**S5**: ANOVA analysis of the fixed effects of the LMM for the physiology parameters. The columns are as follows: Sum of Squares (Sum Sq), Mean Squares (Mean Sq), Degrees of Freedom (NumDF and DenDF), F-values (F value), and p-values (Pr(>F)). Statistical significance is indicated according to the following conventions: *** for p < 0.001, ** for p < 0.01, * for p < 0.05, and a dot (.) for p < 0.1. T_nematode_ and T_water_ stand for nematode and water treatment, respectively.

| Parameter | Factor | Sum Sq | Mean Sq | NumDF | DenDF | F.value | Pr(>F) | Significance |
| --- | --- | --- | --- | --- | --- | --- | --- | --- |
| Photo | T_nematode_ | 5,3928 | 1,3482 | 4 | 202 | 1,6603 | 0,1606 |  |
|  | T_water_ | 29,0399 | 29,0399 | 1 | 202 | 35,763 | 9,97E-09 | *** |
| gsw | T_nematode_ | 2,1792 | 0,5448 | 4 | 202 | 0,731 | 0,5718 |  |
|  | T_water_ | 29,931 | 29,931 | 1 | 202 | 40,158 | 1,5E-09 | *** |
| PhiPS2 | T_nematode_ | 4,6207 | 1,1552 | 4 | 202 | 1,7915 | 0,1319 |  |
|  | T_water_ | 24,9191 | 24,9191 | 1 | 202 | 38,646 | 2,86E-09 | *** |

**S6**: Random effect of the LLM's variance and standard deviation (Std. dev.) for physiology parameters.

| Parameter | Groups | Variance | Std. dev. |
| --- | --- | --- | --- |
| Photo | Date | 0,0637 | 0,2524 |
|  | Residual | 0,812 | 0,9011 |
| gsw | Date | 0,1775 | 0,4213 |
|  | Residual | 0,7453 | 0,8633 |
| PhiPS2 | Date | 0,3423 | 0,5851 |
|  | Residual | 0,6448 | 0,803 |

**S7**: Descriptive statistics of physiological parameters. Summarized at each imaging session (IS 1-3) per separate treatment as mean values with standard deviation (±SD). Abbreviations: Photo – photosynthetic rate [μmol CO_2_ m^-2^ s^-1^], gsw - stomatal conductance [mmol m⁻² s⁻¹], PhiPS2 – effective quantum efficiency of Photosystem II [/]. Irrigation conditions are labeled as "well-watered" (ww) or "water-deficient" (wd). Nematode treatments are labeled with the species - "*G. rostochiensis*" (r), "*G. pallida*" (p), or "control" (c) - and inoculation levels: 0 (none), 1 (low), or 2 (high).

| Treatment | Photo IS1 | Photo IS2 | Photo IS3 | gsw IS1 | gsw IS2 | gsw IS3 | PhiPS2 IS1 | PhiPS2 IS2 | PhiPS2 IS3 |
| --- | --- | --- | --- | --- | --- | --- | --- | --- | --- |
| p.1 wd | 7.7 ± 9.1 | 9.3 ± 3.8 | 10.8 ± 4.7 | 0.148 ± 0.188 | 0.162 ± 0.096 | 0.104 ± 0.047 | 0.164 ± 0.085 | 0.131 ± 0.037 | 0.148 ± 0.038 |
| p.2 wd | 8.7 ± 7.5 | 11.6 ± 3.6 | 11.8 ± 4.2 | 0.148 ± 0.120 | 0.314 ± 0.194 | 0.122 ± 0.063 | 0.166 ± 0.076 | 0.150 ± 0.034 | 0.168 ± 0.031 |
| r.1 wd | 10.3 ± 9.1 | 11.6 ± 2.6 | 12.4 ± 3.7 | 0.210 ± 0.169 | 0.213 ± 0.149 | 0.077 ± 0.064 | 0.203 ± 0.086 | 0.159 ± 0.022 | 0.160 ± 0.048 |
| r.2 wd | 10.8 ± 9.0 | 9.1 ± 2.8 | 10.4 ± 4.8 | 0.241 ± 0.205 | 0.160 ± 0.120 | 0.060 ± 0.058 | 0.199 ± 0.073 | 0.146 ± 0.030 | 0.130 ± 0.035 |
| c.0 wd | 6.3 ± 6.2 | 7.3 ± 4.3 | 9.7 ± 2.3 | 0.099 ± 0.114 | 0.100 ± 0.062 | 0.031 ± 0.032 | 0.152 ± 0.054 | 0.118 ± 0.046 | 0.120 ± 0.031 |
| p.1 ww | 16.8 ± 4.8 | 10.3 ± 2.4 | 13.6 ± 2.3 | 0.340 ± 0.218 | 0.271 ± 0.293 | 0.206 ± 0.075 | 0.260 ± 0.032 | 0.154 ± 0.013 | 0.165 ± 0.044 |
| p.2 ww | 16.6 ± 4.9 | 9.4 ± 4.0 | 13.6 ± 3.4 | 0.378 ± 0.189 | 0.225 ± 0.153 | 0.148 ± 0.103 | 0.269 ± 0.051 | 0.120 ± 0.024 | 0.171 ± 0.042 |
| r.1 ww | 18.1 ± 5.1 | 12 ± 1.7 | 14.3 ± 2.0 | 0.377 ± 0.204 | 0.267 ± 0.169 | 0.129 ± 0.021 | 0.287 ± 0.041 | 0.154 ± 0.020 | 0.179 ± 0.043 |
| r.2 ww | 18.0 ± 3.3 | 9.9 ± 2.7 | 14.5 ± 2.1 | 0.411 ± 0.139 | 0.193 ± 0.137 | 0.141 ± 0.094 | 0.279 ± 0.034 | 0.134 ± 0.027 | 0.182 ± 0.027 |
| c.0 ww | 13.3 ± 4.1 | 12 ± 3.0 | 14.9 ± 1.6 | 0.302 ± 0.174 | 0.297 ± 0.159 | 0.226 ± 0.070 | 0.240 ± 0.033 | 0.154 ± 0.035 | 0.191 ± 0.032 |

**S8:** SHAP-identified most influential spectral bands. Datasets: a) D2 and b) D17. Lighter colors indicate a higher importance of the feature and highlight the spectral channels that are of greater importance for prediction. An accompanying bar chart provides a visual reference for the color shades.


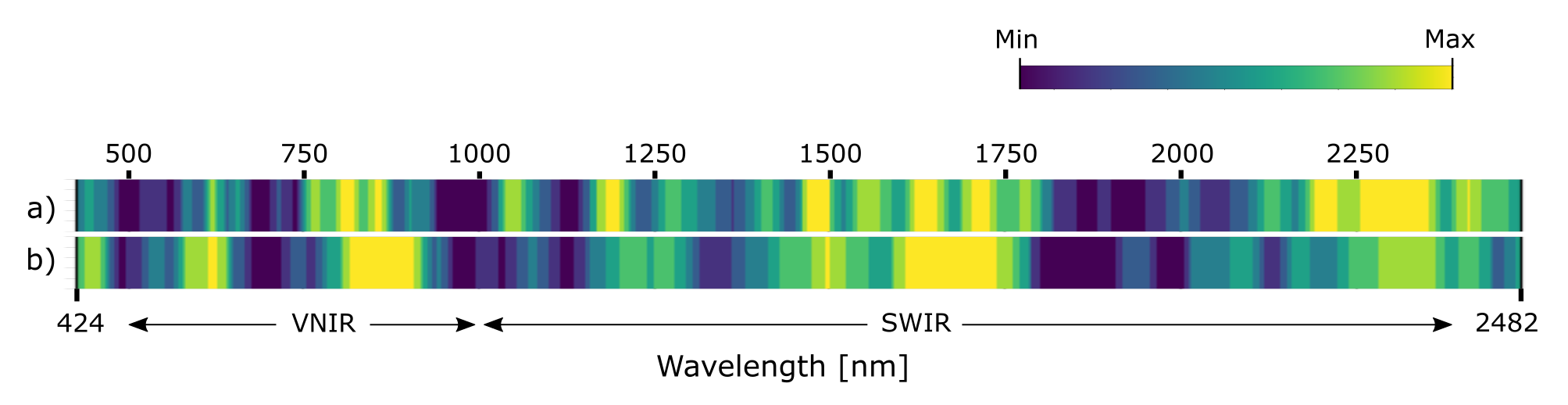


**S9**: Performance of the model with reduced feature set. Datasets: a) D2 and b) D17. The performance of the model is shown as a function of the number of selected spectral bands determined using the SHAP relevance scores. The performance metrics are shown with F1 score in green, precision in blue and recall in orange.


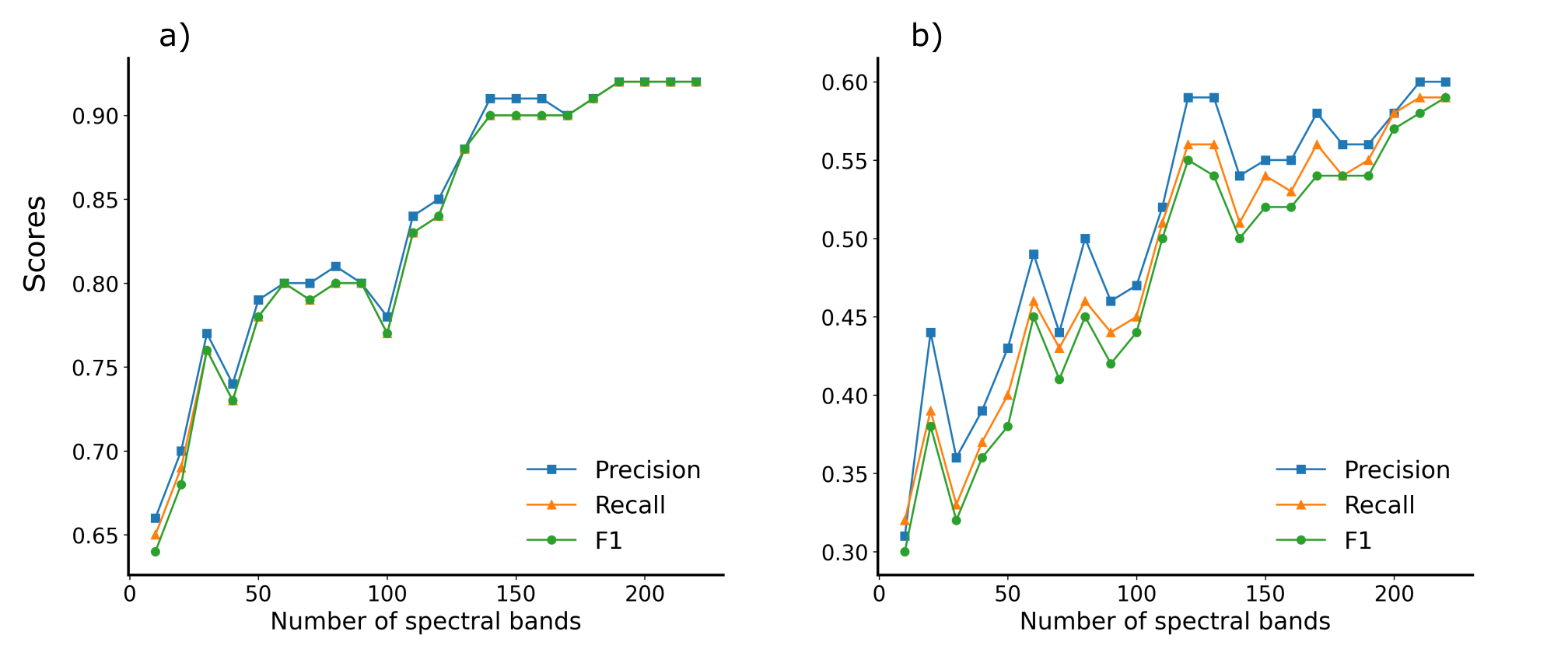

Supplement: Multimedia component 1 [file mmc1.docx]
